# Spectroscopic characterization of charge carrier anisotropic motion in twisted few-layer graphene

**DOI:** 10.1038/srep16388

**Published:** 2015-11-09

**Authors:** Viktor Kandyba, Mikhail Yablonskikh, Alexei Barinov

**Affiliations:** 1 Elettra - Sincrotrone Trieste, s.s. 14 - km.163,5 in Area Science Park, Basovizza, 34149, Trieste, Italy; 2 Physics Department, University of Trieste, Via Valerio 2, 34127, Trieste, Italy

## Abstract

Graphene, a layer of carbon atoms in a honeycomb lattice, captures enormous interest as probably the most promising component of future electronics thanks to its mechanical robustness, flexibility, and unique charge carrier quasiparticles propagating like massless high energy Dirac fermions. If several graphene layers form a stack, the interaction between them is, on the one hand, weak, allowing realization of various registries between the layers and, on the other hand, strong enough for a wide range tuning of the electronic properties. Here we grow few layer graphene with various number of layers and twist configurations and address the electronic properties of individual atomic layers in single microscopic domains using angle-resolved photoelectron spectromicroscopy. The dependence of the interlayer coupling on the twist angle is analyzed and, in the domains with tri-layers and more, if different rotations are present, the electrons in weaker coupled adjacent layers are shown to have different properties manifested by coexisting van Hove singularities, moiré superlattices with corresponding superlattice Dirac points, and charge carrier group velocity renormalizations. Moreover, pronounced anisotropy in the charge carrier motion, opening a possibility to transform strongly coupled graphene bilayers into quasi one-dimensional conductors, is observed.

Unique electronic, structural and mechanical properties of graphene^1^ stimulate much effort in the design of novel devices. The remarkable progress in fabrication achieved recently allows realization of separately contacted layers in few layer graphene (*n*LG)^2^ and control of the twist angle between adjacent layers^3^,^4^, opening a possibility to manipulate properties and thus, the function of the produced device, on a large scale.

When *n* graphene layers have Bernal stacking as in graphite, mirror symmetry plain position depends on parity of *n* and determines the presence of charge carrier massless particles considerably affecting the electronic properties of the system^5^. Significant changes occur also when the rotational symmetry breaks and even a minute twist between the layers can influence the electronic structure and consequently alter the device performance^6^. In fact, the charge carriers propagating like massless Dirac fermions with a group velocity *V*_f_^0^ ~ 1.1 × 10^6^ m s^−1^ in *1*LG^7^ and massive in *2*LG with Bernal stacking^8^,^9^ regain massless behaviour if a twist between the layers is present^10^. After some debate, it was shown that the interlayer coupling, imposing moiré superlattice periodic potential on twisted layers, remains, resulting in the appearance of van Hove singularities (VHS) in the electron density of states^11^,^12^ accompanied by *V*_f_ renormalization^13^,^14^. The position of VHS depends on the twist angle *θ* and for small angles is close to Fermi level (E_f_), leading to peculiar electron localization^11^,^13^,^15^.

In twisted bilayer the graphene layers are equivalent. However, even in this simplest graphene stack, phenomena like counterflow conductivity of separately contacted layers^16^, neutrino-like oscillations and collimated electron transport^17^ are predicted to result from rotational symmetry breaking. These exotic properties are closely related to chiral spinor-like wave function of the quasiparticles in graphene^18^. Moreover, as a distinctive feature of the chirality, the propagation of Dirac fermions under external periodic potential is expected to be anisotropic, manifesting anisotropic group velocities^19^, anisotropic gaps in electronic structure at minizone boundary (MB)^19^ and the formation of superlattice Dirac points^20^.

Experimental view on the anisotropy in the electronic structure of twisted *n*LG is particularly important for small *θ*, for which the above mentioned intriguing phenomena are predicted by tight binding models and at the same time first principle calculations become impractical as the supercell contains big number of atoms. Moreover, the results of *ab initio* calculations for rather large twist angles, suggesting effective decoupling of layers^21^, are in agreement with angle resolved photoelectron spectroscopy (ARPES) from multi-domain samples of *n*LG grown on SiC^22^. However, scanning tunnelling spectroscopy (STS)^11^,^12^,^14^, and recent ARPES^23^ studies as well as our results obtained from *single* domains confirm the presence of interlayer coupling. In the experiment by Ohta and coworkers^23^ *2*LG samples had rather large *θ* (>5^o^) and the domains of ~100 μm size comparable to typical photon beam dimensions in ARPES. The measurements indicate anisotropic gap at MB but non-interacting Dirac cones. Evidence of the velocity renormalization and, hence the interaction of Dirac cones was obtained by Landau level STS in magnetic field^14^, however *k*-resolved density of states and, consequently anisotropy, cannot be addressed by this method.

Here, we use microscopic ARPES (μ-ARPES) of single few micron sized rotationally misoriented domains of twisted *n*LG grown on SiC substrate and illustrate in detail the effects of the interlayer coupling on the electronic structure of individual graphene layers. We show that, when several concomitant couplings break the equivalence between the layers (*n* ≥ 3), the charge carriers may exhibit different transport characteristics in different layers related to a sharp decrease of the interlayer coupling strength as *θ* increases. Besides the presence of coexisting van Hove singularities, moiré superlattice Dirac points, anisotropic gaps at MB and anisotropic *V*_*f*_ renormalizations, we observe non-dispersing band structure regions in strongly coupled graphene bilayers with a small *θ* (<2.7^o^) providing a possibility to transform the bilayers into quasi-one-dimensional conductors by fine tuning of E_f_ position.

## Results and Discussion

**Figure 1:**
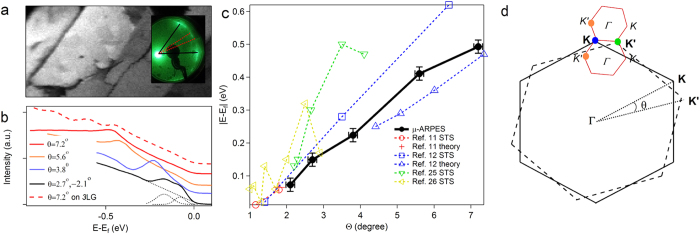
Twisted *n*LG. (**a**) An image of *n*LG surface acquired registering photoelectrons at E ~ E_f_. The **K** point direction of *2d* Brillouin zone of the top layer of brighter (higher count rate) domains corresponds to the electron analyzer orientation^24^. The field of view is 150 × 75 μm^2^. On Low Energy Electron Diffraction pattern acquired at 55 eV (in the inset) main lattice vectors of SiC substrate are indicated by black lines. The electron analyzer acceptance angle for the image acquisition is within red dotted lines. (**b**) Angle integrated spectra from the domains with different twist *θ*. Dotted black curves show decomposition of double VHS spectrum into two components and a linear background. The dashed line is a spectrum from a layer rotated by 7.2^o^ over *3*LG with Bernal stacking showing three VHS peaks (positions are not reported on (c)). (**c**) A summary of VHS peak position *vs θ* (bold line) compared also to STS data and calculations by others (dashed lines). (**d**) First Brillouin zones of two graphene layers rotated with respect to each other (solid and dashed big hexagons) and their common minizones represented by small hexagons. **Γ** is normal to the graphene surface and *Γ* is the centre of the minizone. The coloured circles indicate the region of interest, i.e. measured main **K** (blue), **K’** (green) and supelattice *K’* (orange) Dirac points.

Low Energy Electron Diffraction patterns (Fig. 1a inset) taken from the samples clearly show multiple and elongated diffraction spots reflecting various rotations of graphene domains, although it can be seen that there are also some preferential orientations, for example at 30^o^ with respect to SiC lattice vectors. This rotational misalignment can be directly visualized in the real space using scanning photoelectron microscopy imaging taken with the electron analyzer oriented towards a selected K point^24^. On Fig. 1a various rotational domains of regular shape can be observed. In the following, using angle resolved three-dimensional band structure mapping, we determine *θ* between the layers, the number of layers, velocities of the charge carriers and other important characteristics in individual *n*LG domains (see Methods).

Angle integrated spectra taken from different domains show saddle type VHS peaks shifting towards E_f_ as *θ* decreases (Fig. 1b), similar to the observed by STS^11^,^12^,^25^,^26^,^27^ but only for the occupied electronic states contributing to the photoemission signal. Our results for the positions of the occupied VHS peaks summarized in Fig. 1c are in agreement with theoretical predictions and experimental results of ref. 11 and ref. 12, while some discrepancy can be found if our data are compared with those in ref. 25 and ref. 26. However, in the latter works there is noticeable asymmetry of VHS states with respect to E_f_. This may be related to graphene doping and also effects of the substrate or STM tip, whereas *n*LG domains in the present work are decoupled from the substrate and thus have charge neutrality level or Dirac K-points at E_f_. Note that in the spectrum with *θ = 2.7*^*o*^*,−2.1*^*o*^ the peak is double corresponding to two coexisting VHSs in a *3*LG domain with two different rotations. This spectrum can be decomposed into two peaks after subtraction of a linear background with the positions of corresponding VHS peaks shown on Fig. 1b as two points with the smallest angles in the plot. We also report an angle integrated spectrum for a *4*LG with one layer rotated by 7.2^o^ over *3*LG with Bernal stacking showing three peaks discussed in detail in the last section.

### Observation of *n*LG minizone and main features of electronic structure related to interlayer coupling

A large real space superlattice formed by a pair of twisted layers can be transformed into a minizone^14^,^16^,^27^,^28^,^29^ with *K* and *K’* points originating respectively from the main **K** and **K’** points of the two layers (Fig. 1d). We map *k*-space region around the coloured Dirac points in Fig. 1d, which, as shown in the following, can be easily identified by ARPES.

**Figure 2:**
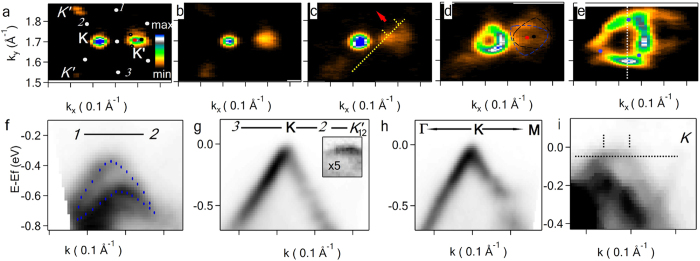
Twisted *3*LG band mapping. μ-ARPES of a *3*LG with *θ* = 1^o^, 5.6^o^ from the bottom layer to the top with the main Dirac points **K** for the top layer and **K’** for the bottom bilayer. *k*-Maps at E = E_f_ (**a**), E = E_f_ − 20 meV (**b**), E = E_f_ − 125 meV(**c**), E = Ef − 300 meV (**d**) and E = E_f_ − 580 meV (**e**) with k_y_ direction along main **ΓK** direction of the top layer and k_x_ perpendicular to it; *k*-E dispersions along *1 – 2* (**f**), 3 - *K’* (**g**) directions indicated in **(a**); (**h**) - along **ΓK** direction or white dotted line shown in (**e**); (**i**) - along yellow dotted line with two crossing lines highlighting the region of a flat band indicated in (**c**). The points in *k*-space where the MB gap is biggest around main Dirac point of the top layer are marked with blue dots in (**e**) whereas blue marks in (**f**) serve as a guide for an eye to show the detailed MB gap evolution. The position of Dirac points the middle and the bottom layers are indicated by respective red and black dots in (**d**), while two black circles in (**a**) indicate the positions of expected *K”* Dirac points of middle-bottom layer superlattice. The intensity to colour conversion at the right of graph (**a**) corresponds also to the graphs (**b–e**), while (**f–i**) are in inverse gray scale. The intensity in the box of (**g**) is multiplied by factor of five.

If *n* ≥ 3, and there are several periodicities when some of the layers experience two different concomitant couplings, the description in terms of a common minizone appears complicated. As we demonstrate below, in many cases the electrons in the superlattices corresponding to different periodicities can be treated independently because of big differences in the coupling strength. Constant energy photoelectron intensity distribution (*k*-map) at E = E_f_ (*I(k*_*x*_*, k*_*y*_*, E = E*_*f*_)) in Fig. 2a shows the main **K**, **K’** and superlattice *K’* Dirac points from a tri-layer domain with the top layer (**K**) rotated by 5.6^o^ with respect to a slightly twisted bottom bilayer (**K’**). The interlayer atomic arrangement in the domain can be considered as superposition of two supercells, one of the bottom and the middle layers and the other smaller (bigger minizone in the reciprocal space) of the top and the middle layer. The presence of a small minizone of the lower bilayer is evidenced at E = E_f_ only by elongated **K’** on Fig. 2a, exhibiting more pronounced supelattice structure on *k*-map at E = E_f_ − 120 meV (Fig. 2c) and two distinct triangle profiles at E = E_f_ − 580 meV highlighted in Fig. 2d. The intensity drop from **K** to **K’** due to attenuation of photoelectrons originating from the bottom layers is used here and below to discriminate the photoemission-related features of different layers, while further decrease from **K’** to *K’* can be rationalized considering that the latter Dirac points are formed as a result of a weak interlayer coupling. *K* points not clearly visible in Fig. 2a are also present but with much lower intensity concluding the formation of the minizone of the top and the middle layers. One can observe faint signatures of *K* points in Dirac spectrum where *K* point is expected (Fig. 2i). We speculate that such difference in photoemission between *K* and *K’* is due to a final state effect in photoemission process, in which the photoelectrons from lower layers experience stronger scattering by the top layer atoms. As a result, experimental *k*-maps follow triangular symmetry and theoretical band structure calculations, having hexagonal representations, are expected to have double number of features if compared to our experimental data.

**Figure 3:**
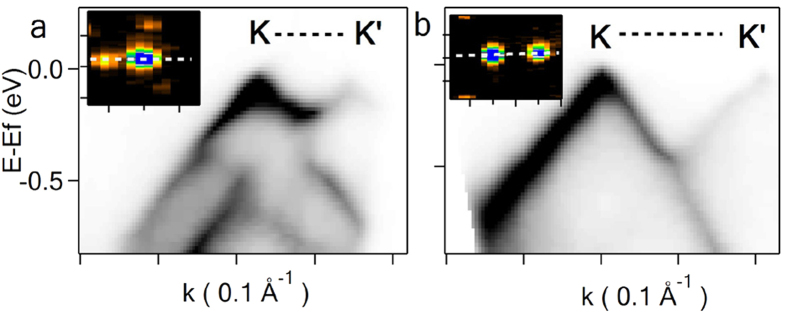
Two types of twisted graphene superlattices. *k*-E dispersions and *k*-maps at E = E_f_ of ‘odd’ and ‘even’ type superlattices with corresponding *θ* = 4.3^o^ (**a**) and 7.2^o^ (**b**). *k*-E dispersion directions of spectra in (**a**,**b**) are indicated by lines in the insets. The tick separation in the insets is equal to 0.1 Å^−1^.

**Figure 4:**
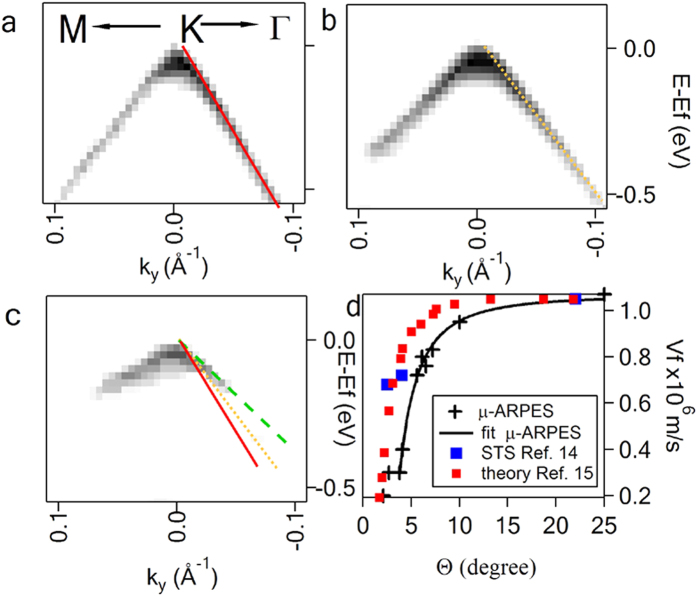
Charge carrier velocity renormalization. *k*-E dispersions at **K** point of the top layer twisted by 25^o^ (**a**), 5.6^o^ (**b**), 4.3^o^ (**c**) with solid, dotted and dashed lines respectively indicating the slopes of the Dirac spectra. Dots with the error bars in (**d**) show *V*_*f*_ obtained from the dispersion relation *E = ћV*_*f*_* k* (μ-ARPES). Solid line in (**d**) is a plot of equation (1) with *t = *95 meV and *V*_*f*_^*0*^ = 1.07 × 10^6^ m/s. For comparison also the data obtained by Landau Level STS and theoretical calculations by others are plotted.

First, we discuss in detail the electrons in the top layer of the above *3*LG and show that all general features of the electronic structure predicted by tight binding calculations and related to the interlayer coupling and ‘spinor’ wave function of Dirac fermions can be clearly resolved: (I) The superlattice gap with the maximum opening of ~175 meV (at blue dots in Fig. 2e) is non-uniform along MB and its evolution can be followed in detail in Fig. 2f. The bands manifest 2/3π rotational symmetry around **K** as shown in Fig. 2e with zero gap along **ΓK**, while clearly open in **KM** (Fig. 2h); (II) The minizone has additional superlattice zero gap points below E_f_. For *θ = *5.6^o^ such points are found in the middle between **K** and **K’**, **K** and *K’* etc., (white dots in Fig. 2a) representing half of the theoretically predicted points for *‘even’* type superlattice^20^. We observe also *‘odd’* type superlattices, e.g. for *θ = *4.3^o^, where the points in the middle between **K** and **K’**, **K** and *K’* correspond to the maximum MB gap opening (Fig. 3a), making characteristic difference between these two types of superlattices with the gap between **K** and **K’** present for the *‘odd’* (Fig. 3a) and absent for the *‘even’* (Fig. 3b) structures; (III) *V*_*f*_ of the charge carriers in the top layer is renormalized from ~1.1 × 10^6^ m/s of isolated graphene to 0.71 × 10^6^ m s^−1^ (Fig. 4b) in quantitative agreement with tight binding model predictions^13^ for a bilayer with *θ* = 5.6^o^.

### Charge carrier velocity renormalization vs twist angle

Dirac spectrum of a layer rotated by a large angle (Fig. 4a) is similar to the spectrum of isolated graphene with *V*_*f*_ = 1.07 × 10^6^ m s^−1^ as measured by ARPES^10^,^30^ and Landau level STS in magnetic field by others^14^,^31^. For *θ* < ~10^o^ the velocity decrease becomes appreciable and can be followed comparing the dispersions in Fig. 4. The absence of the photoemission signal along **KM** for E < E_f_ − 350 meV (Fig. 4b) and in both **KM** and **ΓK** for E < E_f_ − 180 meV (Fig. 4c) is due to the gaps in corresponding MBs. *V*_*f*_^**ΓK**^ in the top layer versus *θ* is summarized in Fig. 4d. Despite the data are obtained from *n*LG domains with different *n*, the results fit with good agreement to tight binding model prediction for a bilayer, with the best fit of the equation *V*_*f*_*/V*_*f*_^*0*^* = 1−9(t/ћV*_*f*_^*0*^*|**ΔK**|)*^2^ (1), for *t* = 95 meV, comparable to calculated *t* = 110 meV, where *t ≈ 0.4t*_*⊥*_ and *t*_*⊥*_ is the interlayer coupling for Bernal stacking^13^. These results are in general agreement with *V*_*f*_ deduced from STS measurements^14^ and theoretical calculations^15^, however with slightly higher velocity renormalization. We tentatively explain this small discrepancy as an effect of electron-phonon coupling leading to observation additional velocity renormalization in photoemission^32^. One can also notice an anisotropy i.e. *V*_*f*_^**ΓK**^ > *V*_*f*_^**KM**^, negligible for *θ = 35*^*o*^ and increasing for small *θ*, which we attribute to stronger interaction of Dirac cones, along **KK’** direction (approximately parallel to **KM**), not observed for large *θ*^23^.

### Electronic structure of a bilayer with a small twist: Fermi surface instability and realization of quasi-one-dimensional conductivity

The middle and the bottom layers of the domain analyzed in Fig. 2 are rotated by only ~1^o^ with respect to each other. Strong interaction of Dirac cones of the layers near E_f_ is manifested by the Fermi surface at **K’** (Fig. 2a) elongated in *k*_***x***_, making difficult to calculate *θ* using the procedure described in Methods. We estimated the twist angle by discerning the two shifted ‘triangular’ profiles of Dirac cones of the two bottom layers on *k*-map well below E_f_ (dashed lines in Fig. 2d), where the interaction between Dirac cones of the layers is not pronounced. In accord with tight binding calculations of the electronic bands, predicting localization and hence flattening of the of electronic bands with the energy close to E_f_ for a bilayer with small *θ*^15^,^16^,^28^,^33^, the bands at **K’** and, accordingly *K’*, have weak dispersion (shown in the box of Fig. 2g).

As can be seen in other *n*LG domains, an equilateral triangle representing the Fermi surface of the underlying layers, surrounding the main Dirac point **K** of the top layer, is a common feature of all superlattices observed by us for different domains. The only exception is the discussed here bilayer, in which the supelattice Dirac points of the bottom layer (*K”* indicated as black circles on Fig. 2a) are absent at E_f_. We tentatively attribute this observation to Fermi surface instability related to charge localization or CDW observed by STS in a bilayer with small *θ*^11^. In fact, CDW instability is expected to remove spectral weight at the band regions around saddle point of VHS and to leave zero gaps, where the Fermi surface is well defined^34^,^35^,^36^. The minizone of the bilayer is too small compared to our *k*-resolution to deduce CDW *Q*-vector in detail and to make any temperature dependent systematic study, but the distortion of the bands near E_f_ observed at the temperature of 110 K used in the experiment can be clearly observed in Fig. 2: the electronic structure of the bilayer gradually develops from non interacting bands (Fig. 2d) through gapped and zero-gap superlattice points at MB (Fig. 2c) into isotropic region (Fig. 2b) followed by elongated Fermi surface (Fig. 2a) indicating that CDW gap is ~20 meV.

**Figure 5:**
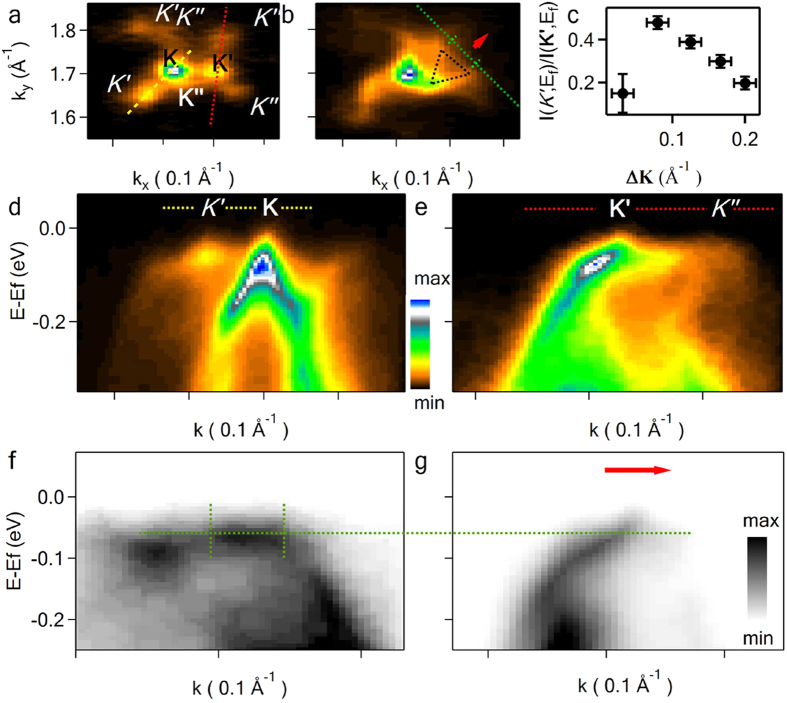
Twisted *3*LG band mapping and the interlayer coupling. μ-ARPES of a *3*LG and a summary of coupling strength vs twist angle. The rotation angle of the middle layer (**K’**) is *θ* = 2.7^o^, with respect to the top layer (**K**) and *θ* = −2.1^o^ with respect to the bottom layer (**K”**). The superlattice points are indicated by *K’* for the superlattice of the top and the middle layer and by *K”* for the superlattice of the middle and the bottom layers; (**c**) a summary of photoelectron intensity ratio I(*K’*,E = E_f_)/I(**K’**, E = E_f_) *vs* the absolute value of **ΔK** = **K**-**K’** for the domains with various rotation angles. *k*-Maps at E = E_f_ (**a**) and E = E_f_ − 80 meV (**b**); *k*-E dispersions for the tri-layer along *K’*-**K** (**d**), ***K’**- K”* (**e**) respective directions shown in (**a**) as yellow and red dotted lines correspondingly, (**f**)-along green dotted line with two crossing lines highlighting the region of a flat band in (**b**,**g**) – along the red arrow in (**b**). The intensity to colour conversion of the graph (**d**) corresponds also to graphs (**a**,**b**,**e**), while the images (**f**,**g**) are in inverse gray scale. The black dotted triangle in (**b**) indicates equivalent directions of *k*-space with flat bands.

Beside the discussed above phenomenon, the relief of electronic bands at a saddle type VHS is expected to result in the appearance of K-E plains with non-dispersing portions of the bands^17^. We observe these regions at E ~ E_f_ − 40 meV within ~0.05 Å^−1^ in *k*-space, (Fig. 2i, between small dotted lines in K-E plain indicated in Fig. 2c), which can be more extended for greater twist angles as shown and discussed also in the following section (Fig. 5f). These non-dispersing band regions allow realization of peculiar transport phenomenon, when a localized hole-like perturbation, 40 meV below E_f_ for the present bilayer with *θ ~ 1*^*o*^, would propagate with a group velocity **V** = ћ^−1^**∇**_**k**_E perpendicular to the flat band in the direction indicated by the arrow and also along its symmetrically equivalent directions. More interestingly, such flat bands can be adjusted to E_f_ by uniform doping of the domain and cause a transition of the graphene bilayer from two-dimensional into a quasi one-dimensional conductor.

### Coupling strength vs twist angle and interplay of several concomitant couplings for n ≥ 3

In *3*LG on Fig. 2, the electronic properties of the top layer are strikingly different compared to the behaviour of slow or even localized electrons in the middle and bottom layers. Summarizing this result we can conclude that on the different ‘floors’ of twisted *n*LG domain the charge carriers can propagate with different group velocities and, if a given layer is found between the two layers with different twist angles, smaller rotation has bigger influence on the electronic structure of the layer. The difference in *θ* of the middle to the bottom and the middle to the top layers for the discussed above *3*LG is big, however this rule holds when the difference in the twist angles is smaller. In the following example of another *3*LG (Fig. 5), despite the absolute values of the rotation angles of the middle layer (**K’**) with respect to the top layer (**K**, *θ* = 2.7^o^) and to the bottom layer (**K”**, *θ* = −2.1^o^), are similar, there is considerable difference in the corresponding VHS peak positions at E ~ E_f_ − 150 meV and E ~ E_f_ − 73 meV respectively (Fig. 1b). The group velocities of the charge carriers in the layers are also different with *V*_*f*_  ≈ 0.2 × 10^6^ m/s for the bottom and the middle layers, whereas *V*_*f*_ ≈ 0.35 × 10^6^ m/s for the top layer (Fig. 5d,e). The behaviour of the electrons in the middle layer is essentially more affected by the interaction with the bottom layer than with the top layer rotated by greater angle with respect to it. Indeed, the electronic band along **K’** - **K”** branWch is symmetric with similar *V*_f_ in both the middle and the bottom layers (Fig. 5e). This result indicates that apparently, a minute change in *θ* dramatically affects the interlayer coupling. In fact, the interlayer tunnelling term t(Δk) in tight binding calculations rapidly drops to zero with the increase of transfer momentum Δ**K**^16^,^37^,^38^,^39^. In Fig. 5c we plot the ratio of the photoelectron intensity at E_f_ for the superlattice Dirac point *K’* to the intensity at the main Dirac point **K’** I(k = *K’*, E = E_f_)/I(k = **K’**, E = E_f_) that can be used as a quantitative measure of the relative coupling strength. Its quick decrease as Δ**K** increases illustrates the sharp dependence of the interlayer coupling on *θ*. However, the ratio drops for small angles as shown for Δ**K** = 0.032 Å^−1^ representing ~1^0^ twist from the previous section. This drop in the ratio can be associated with the discussed above CDW instability. We obtained I(k = *K’*, E = E_f_)/I(k = **K’**, E = E_f_) < 0.23 by fitting I(k, E = E_f_) of Fig. 2a in the region around **K’** with four *2d* gaussians representing two main (red and black dots in Fig. 2a) and two *‘expected’* (black circles in Fig. 2a) superlattice Dirac points situated in a similar fashion as **K**, **K’**, *K’* of the big minizone. However, the actual ratio can be smaller or even zero since the experimental energy resolution is comparable to CDW gap.

Also in the minizone formed by the middle and the bottom layers of this domain we observe flat non dispersing bands (Fig. 5f). The group velocity of the excitation close to corresponding VHS peak, i.e. at E ~ E_f_ − 60 meV, is directed along the red arrow perpendicularly to **K’** - **K”** and its equivalent directions (dotted triangle in Fig. 5b) and can be estimated ~1.5 × 10^5^ m s^−1^ from the corresponding K-E dispersion in Fig. 5g. We could find clear evidence of the flat bands, which may be used to realize one-dimensional transport, in the minizones corresponding only to small *θ* such as 1.1^o^ and 2.1^o^ presented in the paper. In both cases E_f_ shift needed for switching to quasi-one-dimensional conductivity is less than 100 meV and can be achieved by small doping level below 0.003 e^−^ per unit cell, not changing bilayer electronic structure if each layer is doped by the same amount^8^.

**Figure 6:**
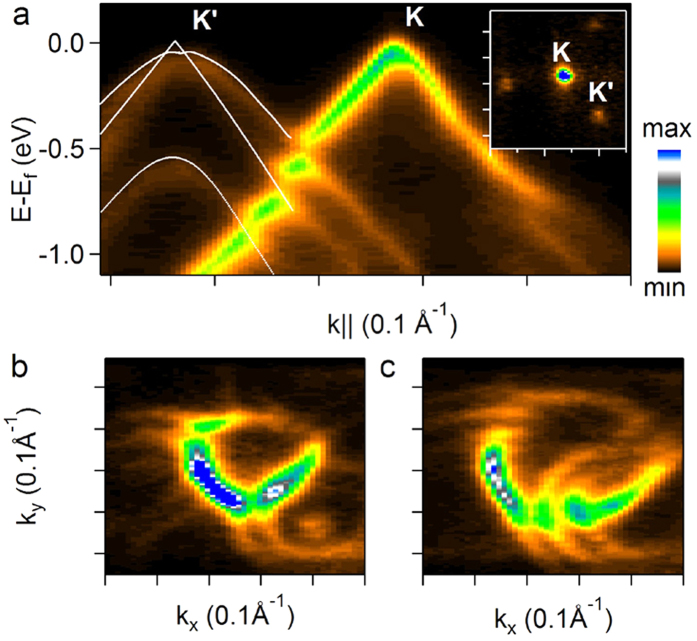
Band mapping of graphene layer (K) rotated by *θ* = 7.2^o^ with respect to a *3*LG with Bernal stacking (K’). *k*-E dispersion (**a**) along **K- K’** direction and *k*-map at E = E_f_ shown in the inset. The lines in (**a**) is the result of tight binding calculations for *3*LG with Bernal stacking. *k*-Maps at E = E_f_ − 0.7 eV and E = E_f_ − 1 eV are plotted in (**b**,**c**) respectively. The tick separation in the inset of (**a**) is equal to 0.1 Å^−1^.

Finally, we note that stronger coupling delocalizes the electronic bands between the layers and makes another twisted weakly coupled layer to interact with such system as a whole. To demonstrate this interaction we present an extreme case: a layer rotated by 7.2^o^ on top of *3*LG with Bernal stacking (Fig. 6), representing the strongest interlayer coupling in *n*LG. Near E_f_, the electronic structure of the underlying tri-layer is composed of three common electronic bands, two reaching E_f_ and one parabolic with a maximum at E = E_f_ − 0.5 eV. Having odd number of layers, the carriers in one of the two bands at E_f_ are massless^5^. These bands are described with good agreement by tight binding Hamiltonian and overlap parameters taken from ref. 40. Good correspondence suggests that electronic structure of the *3*LG is not perturbed by the presence of the fourth rotated layer on top, while the top layer on the contrary has clear signatures of its interaction with the underlying *3*LG domain. Indeed, there are three gaps in the minizone of the top layer (Fig. 6b,c) and three VHS peaks in the angle integrated spectrum (Fig. 1b) corresponding to each of the three electronic bands of the underlying tri-layer. Also *V*_*f*_ at **K** is renormalized to 0.85 × 10^6^ m s^−1^. Interestingly, the data taken from the same top layer but from its part overlaying a *1*LG with the same twist are presented in Fig. 3b. In this case there is only one VHS peak (Fig. 1b) and *V*_*f*_ of the underlying layer in Fig. 3b is also renormalized, which is not the case in the massless carriers branch of the bottom tri-layer in Fig. 6. The superlattice potential created by *3*LG is apparently different from a single layer showing no gap opening in **K**-**K’** direction characteristic for even type superlattice. The lower energy VHS position for the layer rotated by 7.2^o^ over *3*LG is closer to E_f_ than VHS position for the same rotation over *1*LG by ~30 meV (Fig. 1b), in qualitative agreement with the results of comparison for a layer rotated over *1*LG and *2*LG with Bernal stacking^27^. However, the difference in VHS peak energies is too small to expect appreciable variation in *V*_*f*_ in the top layer in these two cases.

In conclusion, the rich variety of electronic structure phenomena giving a possibility of a wide range tuning of the electronic structure of twisted few layer graphene is observed. In the presence of several interlayer couplings the electronic properties can be completely different in different layers of the same domain providing independent channels of electron transport. The group velocity of the charge carriers propagating in the moiré potential of strongly coupled (slightly twisted with *θ* < 2.5^o^) layers is not only renormalized, but can be anisotropic, giving unique possibility to transform the graphene from the two-dimensional into a quasi-one-dimensional conductor using two tuning parameters: E_f_ (by doping) and *θ*. In the case of smaller twist angles (~1^o^), when the electron localization is expected, the electronic bands become weakly dispersing and hint on the appearance of CDW instabilities. We believe that the obtained results can further stimulate the device development, in which different layers may have separate electric contacts and the electronic properties of the graphene ‘floors’ can be engineered by their respective twists.

## Methods

The experiment was performed using 27 eV photon beam from synchrotron radiation undulator beamline 3.2L of Elettra light source producing linearly polarized light (along k_x_ direction) focused to ~0.6 μm spot with Schwarzschild objective^24^ and incident at 45^o^ with respect to the sample surface. ARPES data were acquired with a hemispherical electron energy analyzer registering the photoelectrons with total energy resolution of ~30 meV and angular resolutions of ± 0.15^o^ along the angular dispersion direction of the analyzer (k_y_ direction) and ± 0.33^o^ across (k_x_ direction). The sample was mounted on a scanning stage, which enables positioning and raster imaging with respect to the fixed photon beam. *3d* photoelectron intensity distribution maps *I(k*_*x*_*,k*_*y*_*,E)* from the microscopic areas were taken by rotating the electron energy analyzer with respect to the sample, using a two-axis goniometer.

The samples, n-type (5 × 10^16^cm^−3^) C-face 6H-SiC single crystal *(0, 0, 0, 1)* oriented surfaces polished to < 1 Å RMS (Novasic) were first resistively heated in vacuum up to 1000^o^C to clean the substrates and finally annealed in 1 bar of Ar at 1350–1500^o^C for several minutes to obtain *n*LG flakes. *n*LG was prepared in a separate chamber directly connected to the measurement station and subsequently transferred for the measurements without vacuum loss. Whereas the graphene growth on Si-face SiC is epitaxial, for C-face in Ar atmosphere few micron sized domains of various rotational compositions between the layers are grown^41^. In accord with the scope of the present research, we were searching and comparing the results for the domains with various twists between the layers, while also the domains with Bernal stacking can be found as in ref. 42.

Dirac points, i.e. the crossing energy positions of Dirac conical spectra in graphene, are found at hexagon **K** vertexes of two-dimensional Brillouin zone readily accessible with ARPES^10^, and *θ* between the adjacent layers can be evaluated in the reciprocal space directly from Δ**K** = **K**-**K’** (Fig. 1d). |***ΔK**| = 2|***ΓK|***Sin(θ/2)* and **ΓK** is the principal vector of graphene Brillouin zone (|**ΓK**| *≈ 1.7 Å*^−*1*^). The number of layers in a particular domain is equal to the number of main non-equivalent **K** points (**K**, **K’**, **K”** as in Figs 2, 3 and 5) or, for Bernal stacking, is inferred by the number of electronic π-bands^5^,^8^,^40^,^42^. For example, for the domain of *4*LG in Fig. 6 there are two main **K** points and to one of them correspond three π-bands.

In order to select the rotational domains, the images (Fig. 1a) were taken by registering the photoelectrons at E_f_ within ± 250 meV and ± 5^o^ angle windows^24^ keeping the analyzer at 43^o^ with respect to the sample normal corresponding to photoemission from |**ΓK|**. In this case, the domains with the top layer oriented with **K-**point in the same direction as the analyzer give stronger contribution to the intensity, whereas the gradations of intensity on the image reflect the relative rotational orientation of the domains.

The constant energy photoelectron intensity distribution surfaces (*k*-maps) were obtained from *I(k*_*x*_*,k*_*y*_*,E)* within the energy window of ± 10 meV. *V*_*f*_ of the charge carriers was estimated using linear dispersion relation for Dirac fermions E = ћ*V*_*f*_K, where K is the absolute value of the wave vector measured with respect to Dirac point.

## Additional Information

**How to cite this article**: Kandyba, V. *et al*. Spectroscopic characterization of charge carrier anisotropic motion in twisted few-layer graphene. *Sci. Rep*. **5**, 16388; doi: 10.1038/srep16388 (2015).
